# HDAC1 inhibition ameliorates TDP-43-induced cell death in vitro and in vivo

**DOI:** 10.1038/s41419-020-2580-3

**Published:** 2020-05-14

**Authors:** Simona Sanna, Sonia Esposito, Alessandra Masala, Paola Sini, Gabriele Nieddu, Manuela Galioto, Milena Fais, Ciro Iaccarino, Gianluca Cestra, Claudia Crosio

**Affiliations:** 1 Department of Biomedical Sciences, University of Sassari, Via Muroni 25, I-07100 Sassari, Italy; 2 Istitute of Molecular Biology and Pathology-National Research Council at Department of Biology and Biotechnology-Charles Darwin, Sapienza University of Rome, P.Le A.Moro 5, I-00185 Rome, Italy

**Keywords:** Epigenetics, Amyotrophic lateral sclerosis

## Abstract

TDP-43 pathology is a disease hallmark that characterizes both amyotrophic lateral sclerosis (ALS) and frontotemporal lobar degeneration (FTLD-TDP). TDP-43 undergoes several posttranslational modifications that can change its biological activities and its aggregative propensity, which is a common hallmark of different neurodegenerative conditions. New evidence is provided by the current study pointing at TDP-43 acetylation in ALS cellular models. Using both in vitro and in vivo approaches, we demonstrate that TDP-43 interacts with histone deacetylase 1 (HDAC1) via RRM1 and RRM2 domains, that are known to contain the two major TDP-43 acetylation sites, K142 and K192. Moreover, we show that TDP-43 is a direct transcriptional activator of CHOP promoter and this activity is regulated by acetylation. Finally and most importantly, we observe both in cell culture and in Drosophila that a HDCA1 reduced level (genomic inactivation or siRNA) or treatment with pan-HDAC inhibitors exert a protective role against WT or pathological mutant TDP-43 toxicity, suggesting TDP-43 acetylation as a new potential therapeutic target. HDAC inhibition efficacy in neurodegeneration has long been debated, but future investigations are warranted in this area. Selection of more specific HDAC inhibitors is still a promising option for neuronal protection especially as HDAC1 appears as a downstream target of both TDP- 43 and FUS, another ALS-related gene.

## Introduction

TDP-43 or TARDBP (TAR-DNA binding protein-43) is a 43 kDa ribonucleoprotein, originally identified as transcriptional repressor of HIV1 TAR-DNA, associated during the past decades with a spectrum of neurological diseases, namely TDP-43 proteinopathy^1^. TDP-43 is a predominantly nuclear RNA/DNA-binding protein essential for the development of the central nervous system (CNS) from the earliest stages of embryonic life to adulthood^2^. In the nucleus, TDP-43 is involved in transcriptional regulation, splicing and miRNA biogenesis, but upon stressing conditions it is partially relocated in the cytoplasm, becoming involved in mRNA stability control, translation, and nucleocytoplasmic transport by forming stress granules^3^. TDP-43 undergoes several posttranslational modifications that can change its structure, localization, overall functions, its aggregative propensity and, importantly, TDP-43-positive aggregates are a common hallmark of different neurodegenerative conditions^4^. Ubiquitinated, phosphorylated, and acetylated-TDP-43 aggregates were, in fact, identified in 95–97% of patients affected by amyotrophic lateral sclerosis (ALS) and in about 50% of Frontotemporal Lobar Degeneration (FTLD)^1,5,6^. Moreover, the direct role of TDP-43 in disease pathogenesis is underscored by the identification of more than 40 ALS-associated dominant missense mutations in the TDP-43 gene (*TARDBP*) in both familial and sporadic patients^7^. Several different pathological mutants display a propensity to form both nuclear and cytoplasmic aggregates indicating that loss of TDP-43 homeostasis and aggregation play a critical role in pathogenesis. In particular it has been demonstrated that acetylation modifies both TDP-43 function and localization, by decreasing its ability to interact with nucleic acids^5,8^. According to Choen et al.^9^ TDP-43 can be acetylated mainly on K154 and K192 by the HAT (histone acetyl transferase) activity of CBP (CREB binding-protein). Once acetylated, TDP-43 can be relocated in the cytoplasm where it can be deacetylated by the histone deacetylase 6 (HDAC6)^9,10^. Given this experimental evidence, it is plausible that modulating TDP-43 acetylation might be a good strategy to prevent TDP-43 aggregation and cell damage.

HDACs are a complex family of proteins involved in many different cellular functions in CNS, including chromatin shaping to adjust transcriptional profiles during neuronal development and neuronal response to injury^11^. HDAC substrates, which are implicated in neuronal development and survival, are not restricted to histones. As demonstrated, several different physiological functions are regulated by deacteylation^12–14^. Among the different HDACs functionally associated with ALS onset and progression we focused our attention on HDAC1. HDAC1 is a Zn+-dependent deacetylase of 482 amino acids, that can be found in large transcriptional repression complexes consisting of SIN3A, NuRD, and Co-REST^15^, which inactivate the expression of neuronal genes in nonnervous tissues^16^. The role of HDAC1 in regulating neuronal viability is quite controversial ranging from neuronal protection from death to an increase in neurodegeneration and axonal death^12^. The involvement of class I HDACs in the onset of ALS was first shown by Janssen et al.^17^. Different studies also suggest that some of these HDACs regulate vitality and mortality of nerve cells; first, HDAC1 assumes neurotoxic or neuroprotective function as it interacts with HDAC3 or HDAC9^18^. In fact, the role of HDAC1 in regulating neuronal vitality is quite controversial, since some studies show that such deacetylase is able to protect neurons from death, while other studies demonstrate that HDAC1 induces neurodegeneration and axonal death^18,19^. Moreover, recent studies indicate that in degenerative neurons, HDAC1 moves to cytoplasm where it becomes implicated in axonal alteration and degeneration of the cell^19^.

Most relevant to ALS, HDAC1 interacts with FUS, another ALS-causative gene, on DNA double strand breaks and this interaction seems to be important for chromatin integrity; as a matter of fact, many FUS pathological mutations impair this interaction and lead to impaired DNA break repair^20,21^. Notably, ALS patients show increased levels of ROS but also of 8-hydroxy-2 ‘-deoxyguanosine (OH 8dG), a marker of DNA damage. In this context, the interaction between FUS (but also TDP-43) and HDAC1 could have an important role in preserving DNA stability and cell survival, and the alteration of this interaction in ALS patients could lead to an imbalance in the delicate equilibrium between cell survival and cell death. Recently also TDP-43 has been shown to play a key role in DNA damage response (DDR), since its loss of function results in a faulty repair of DNA damage associated with a stopping in transcription and an inhibition of recruitment of critical components of the NHEJ repair system^22,23^.

The striking functional and structural similarities between TDP-43 and FUS^24^, as well as the observation that acetylation appears to promote aggregation and diminish TDP-43 functionality^10^, prompted us to investigate a possible interaction between HDAC1 and TDP-43. Since epigenetic drugs are at present one of the most promising strategies for ALS treatment, information on the physical, and functional interaction between TDP-43 and HDAC1 will provide the rationale for using the HDAC inhibitor (HDACi) in ALS therapy^25^.

## Results

### TDP-43 interacts with HDAC1 in vitro and in vivo, via RNA binding domains

**Fig. 1:**
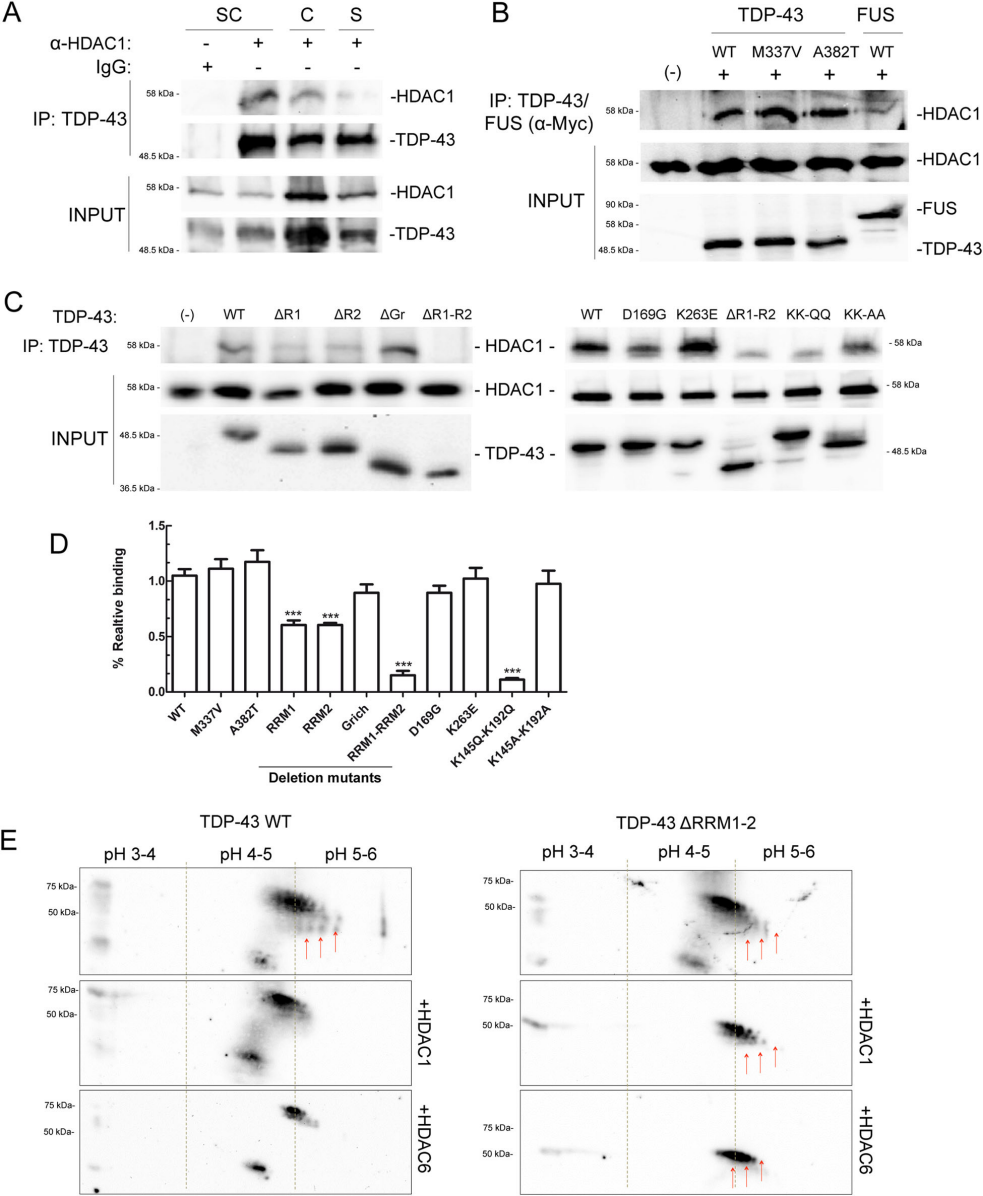
TDP-43 interacts in vivo and in vitro via RRM1 and RRM2 domains, with HDAC1 that can module its acetylation. **a** Spinal cord, cerebellum, or striatum of BALB31c mice were used for co-immunoprecipitation experiments. After dissection tissues were lysed and protein extracts were immunoprecipitated, with specific antibodies. Proteins retained were separated on SDS-PAGE and visualized by western blot using specific antibodies (**b**, **c**) HEK 293T cells were transiently transfected with FLAG-tagged HDCA1 and Myc-tagged WT or mutant TDP-43. We tested pathological mutants M337V, A382T, D169G, K263E, acetylation null KK-AA or mimicking KK-QQ mutants, and the deletion mutants ΔRRM1, ΔRRM2, ΔG-rich, ΔRRM1/RRM2. Forty-eight hours after transfection, cells were lysed, protein extracts were immunoprecipitated and analysed as in (**a**). **d** Bar graph shows the relative binding of HDAC1 to mutant TDP-43, normalized to TDP-43 WT. The data were obtained from four independent experiments; *p > 0.05 and **p > 0.01 versus WT binding, analysed by using one-way ANOVA with Bonferroni’s Multiple comparison post-hoc test. **e** Representative 2-DE maps showing the change in TDP-43 acetylation status, upon HDAC1 or HDAC6 expression. SH-SY5Y were transfected by TDP-43 WT or deletion mutant ΔRRM1-2 and FLAG-tagged HDCA1 or HDAC6. Forty-eight hours after transduction, TDP-43 was immunoprecipitated and a visualized by western blot using an anti-acetyl lysine antibody.

To assess the interaction between TDP-43 and HDAC1 a co-immunoprecipitation assay from mice neuronal tissues was performed, using an anti-TDP-43 antibody. As illustrated in Fig. 1a, a strong interaction between the two proteins in different neuronal tissues was observed, especially in the spinal cord. By transfection and co-immunoprecipitation this interaction was dissected and it was observed that TDP-43 binds to HDAC1 independently from the presence of the pathogenic point mutations M337V or A382T (Fig. 1b). Furthermore, this interaction was compared with the interaction between FUS and HDAC1 described elsewhere^20^ showing a more prominent binding of HDAC1 to TDP-43 compared with FUS. Since HDAC1 and HDAC2 exhibit redundancies in various systems, a co-immunoprecipitation experiment was performed demonstrating no interaction between HDAC2 and TDP-43 (Fig. S1b).

To characterize TDP-43 domain(s) responsible for the interaction with HDAC1 a series of Myc-tagged TDP-43 fragments was generated, lacking the various putative functional domains of the protein (N-terminal, RRM1, RRM2, and G-rich domain). Since the N-terminal deletion causes the complete cytoplasmic re-localization of TDP-43 (Fig. S1), while HDAC1 is mainly nuclear, this mutant was excluded from further analysis. Co-immunoprecipitation experiments, performed on cell lysates from transfect cells, demonstrated that TDP-43 interacts with HDAC1 via both RRM1 and RRM2 domains, and that only in the double deletion mutant the interaction is abolished (Fig. 1c). Interestingly, RMM1 and RRM2 domains have been shown to be crucial in TDP-43 physiopathology^9,26^. Two of the three TDP-43 pathological mutants that do not target the G-rich domain, ALS-linked mutation D169G^27^, and FTLD-TDP linked mutation K263E^28^ map in this region, as well as the two major TDP-43 acetylation sites, K145 and K192^9^. Thus, constructs coding for these pathogenic (D169G, K263E) or acetylation-mimic (KK-QQ) or acetylation-null (KK-AA) mutations were generated to assess whether they can affect the interaction with HDAC1. They were transiently expressed in SH-SY5Y cells and their localization was analysed by immunofluorescence (Fig. S1) demonstrating that all these variants are exclusively nuclear with the exception of TDP-43 D169G, which partially localizes in the cytoplasm. Moreover, according to previous published results, ΔRRM1-2 deletion mutant appeared more clustered in nuclear bodies, which were larger and more numerous compared with TDP-43 WT.

Interestingly, in the co-immunoprecipitation experiments performed the acetylation-mimic mutant (KK-QQ) displays a significant decrease in HDAC1 binding (Fig. 1c, d).

### HDCA1 modulates TDP-43 acetylation

To study the interaction between TDP-43 and HDAC1, first their subcellular localization in SH-SY5Y cells was analysed. By immunofluorescence staining it was demonstrated that cellular stress induced by the overexpression of different TDP-43 mutants does not significantly affect HDAC1 localization (Fig. S2).

Up to now, acetylated-TDP-43 has been demonstrated to be only an HDAC6 substrate^9^. In order to investigate if HDAC1 alters TDP-43 acetylation, we performed a 2D gel-analysis of immunoprecipitated TDP-43 from SH-SY5Y cells transfected with TDP-43 alone or in combination with HDAC1. After immunoprecipitation TDP-43 was separated by isoelectrofocusing and SDS/PAGE. The level of TDP-43 acetylation was evaluated using anti-acetyl lysine antibody (Fig. 1e). The co-transfection with HDCA1, as well as the one with HDAC6, shifts the isoelectric point (pI) of immunoprecipitated TDP-43. This effect is partially prevented by deletion of RRM1-RRM2 domains, confirming that K145 and K192 are prominent but nor exclusive acetylation sites^9^ and suggesting that also HDAC1 can modify TDP-43 pI, most likely by removing acetyl groups.

### TDP-43 activates CHOP transcription and it is regulated by acetylation

**Fig. 2:**
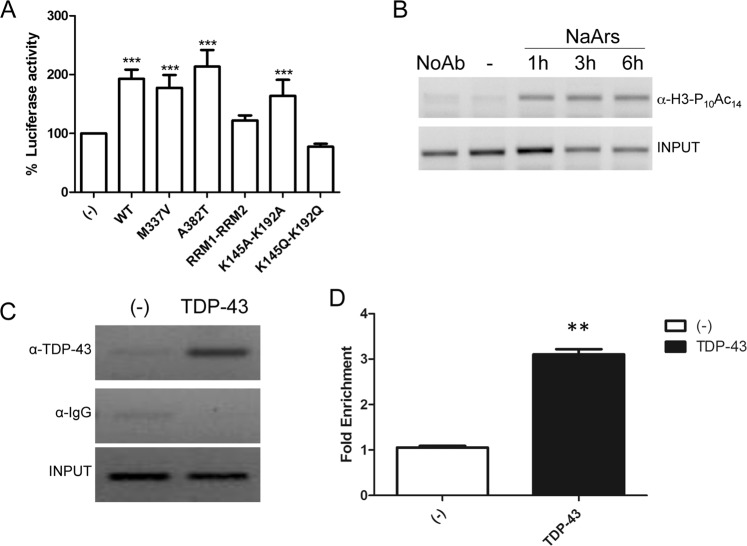
TDP-43 transcriptional activity on CHOP promoter. **a** CHOP-luciferase plasmid was co-transfected with WT or mutant TDP-43 in SH-SY5Y cells and luciferase activity was measured, in a multiplate reader using the Dual-GlowTM Luciferase Assay System (Promega, USA). Firefly luciferase activity was then normalized to the Renilla luciferase activity to control the transfection efficiency. Data were then normalized to luciferase activity in cells transfected with empty vector, which was given a value of 100%. The data were obtained from four independent experiments; **p > 0.01 and ***p > 0.001, analysed by using one-way ANOVA with Bonferroni’s Multiple comparison post-hoc test. **b** ChIP assay by anti-H3-PS10- AcK14H3 on CHOP promoter upon treatment with sodium arsenite (10 µM) for 1, 3, or 6 h, using EZ-Magna ChIP™ (Millipore), according to the manufacturer’s protocol. **c** SHSY5Y cells were transduced with TDP-43 and 24 h postinfection cells were fixed, lysed and used for ChIP analysis. **d** quantification of ChIP experiment by using the Fold Enrichment Method. The data were obtained from four independent experiments; **p > 0.015 for the fold enrichment, analysed by using one-way ANOVA with Bonferroni’s Multiple comparison post-hoc test.

TDP-43 was originally described as transcription factor for TAR DNA of HIV1^29^, but at present the only direct other target is the testis specific mouse acrv1 (SP10) promoter^30,31^. TDP-43 has been also shown to induce a transcriptional upregulation of C/EBP-homologous protein (CHOP) promoter and CHOP gene disruption markedly attenuates TDP-43-induced cell death^32^. Moreover, TDP-43-induced upregulation of CHOP expression is mediated by both reduction of CHOP degradation and by the increase of CHOP mRNA level. Thus, we decided to use CHOP promoter (from −954 to +91) to drive the expression of luciferase reporter and to test the ability of TDP-43 in regulating it. As shown in Fig. 2a, TDP-43 acts as a robust activator of CHOP promoter and the transcriptional activation on this promoter is slightly increased by the overexpression of the pathological mutation A382T, although it does not reach a statistical significance; notably, the transcriptional activation is abolished by RRM1–RRM2 deletion and particularly by the acetylation-mimic point mutations (KK-QQ).

This experimental evidence indicates a direct interaction between TDP-43 and CHOP promoter, which was confirmed by the ChIP approach. In a first attempt, the CHOP promoter activation was tested in response to sodium arsenite using the dual modification of histone H3, which is phosphorylated at serine 10 and acetylated at lysine 14 (H3-PS10/AcK14), as a marker of transcriptional activation. Accordingly, we observed that sodium arsenite treatment induces CHOP transcriptional activation demonstrated by histone H3 phosho-acetylation detection (Fig. 2b). Subsequently, chromatin was extracted and immunoprecipitated using anti-TDP-43 antibodies from SH-SY5Y cells transduced with adenoviral particles expressing 5xMyc-TDP-43^33^. As shown in Fig. 2c, d, we demonstrate that TDP-43 binds directly to the CHOP promoter, in a region comprised between −300 and −30.

### Acetylation affects TDP-43 re-localization under stressful conditions

**Fig. 3:**
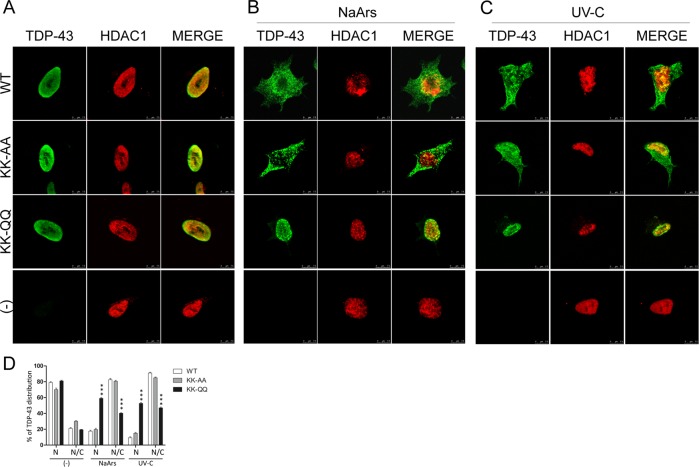
Effect of NaArs or UV-C exposure on TDP-43 localization in SH-SY5Y cells. **a** Immunofluorescence on SH-SY5Y cells transduced with the indicated adenoviral particles encoding for TDP-43 WT, KK-AA, or KK-QQ (MOI 10 pfu/cell), and exposed to NaArs 50 µM for 16 h or UV-C 5 J. The TDP-43 signal was revealed by primary anti-TDP-43 antibodies and anti-mouse ALEXA 488 secondary, HDAC1 was detected by anti-HDAC1 antigen and secondary anti-rabbit ALEXA 647. The slides were analysed by Leica SP5 confocal microscope. **b** Graph summarizing TDP-43 localization (N nucleus or N/C nucleus-cytoplasm) in 200 cells in a total of three different experiments, in the indicated experimental conditions; ***p > 0.001 analysed by using two-way ANOVA with Bonferroni’s Multiple comparison post-hoc test.

TDP-43 acetylation status is suggested to be critical for TDP-43 toxicity, since the TDP-43 acetylation-mimic mutant K145Q induces TDP-43 pathology in muscle cells^10^. Starting from this observation the effects of double acetylatyion mimic or acetylation-null mutations on TDP-43 toxicity were assessed by measuring TDP-43 nuclear loss and cell death of SH-SY5Y cells^34^. In particular, SH-SY5Y transduced with TDP-43 WT or bearing different mutations, were exposed to sodium arsenite, a classical agent used to induce stress granules formation, or UV-C to induce DDR^35,36^. Although both treatments induce the re-localization of WT or acetylation-null mutations in the cytoplasm (Fig. 3), only the acetylation-mimicking KK-QQ mutant is completely unaffected and retained in the nucleus of treated and untreated cells.

### TDP-43 and HDAC1 have a synergistic effect in decreasing cell vitality

The overexpression of either WT or pathological mutants of TDP-43 induces a reduction in cell survival, in different cellular, and animal systems^37^, including SH-SY5Y cells^33^. Thus, the effect of HDAC1 on TDP-43-induced cell toxicity was evaluated. Firstly, adenoviral particles expressing WT or mutant TDP-43 variants were generated and their effects on cell viability were measured. As shown in Fig. S4a, at MOI of 10 pfu/cell, the expression of WT or pathological mutant TDP-43 induces a decrease in cell viability. Notably, at the same MOI the expression of the KK-QQ mutant is less toxic, while the KK-AA mutant displays an intermediate effect. In this condition the ratio between endogenous and exogenous TDP-43 (corresponding to Myc-tagged TDP-43), measured by relative quantification of protein bands, is about 1:5 (Fig. S4b). To better characterize the molecular mechanisms underlying the reduction in cell viability that we observed by MTS assay upon TDP-43 overexpression we evaluated the expression of apoptotic (caspase-3, PARP) or autophagic (LC3) markers, in SH-SY5Y and SH-SY5Y cells stably expressing CFP-DEVD-YFP reporter^38^. As summarized in Fig. S4c, d, the reduction on cell viability likely does not involve the activation of apoptotic or autophagic pathways.

HDAC inhibition has been shown to be protective in a wide range of pathological conditions^39,40^, including ALS. HDACis such as sodium butyrate (NaB), 4-phenylbutyrate (SPB), trichostatin A (TSA), and fatty acid derivatives, which inhibit most class I and II HDACs, have been used in SOD1-G93A mouse model. In fact, SPB treatment extends survival and motor performance in SOD1-G93A mice models and it has been demonstrated to be safe and well tolerated^41,42^ in a phase 2 clinical trial. TSA induces a modest improvement in motor function and survival as well as protection against motor neuron death^43^. Furthermore, butyrate and valproic acid are also known to readily cross the BBB^44^. Finally, the therapeutic potential of NaB has been also demonstrated in other neurodegenerative diseases such as Alzheimer disease, Parkinson’s and Huntington disease^40^. Thus, the effects of these four HDACis on TDP-43-mediated cell death were assessed.

**Fig. 4:**
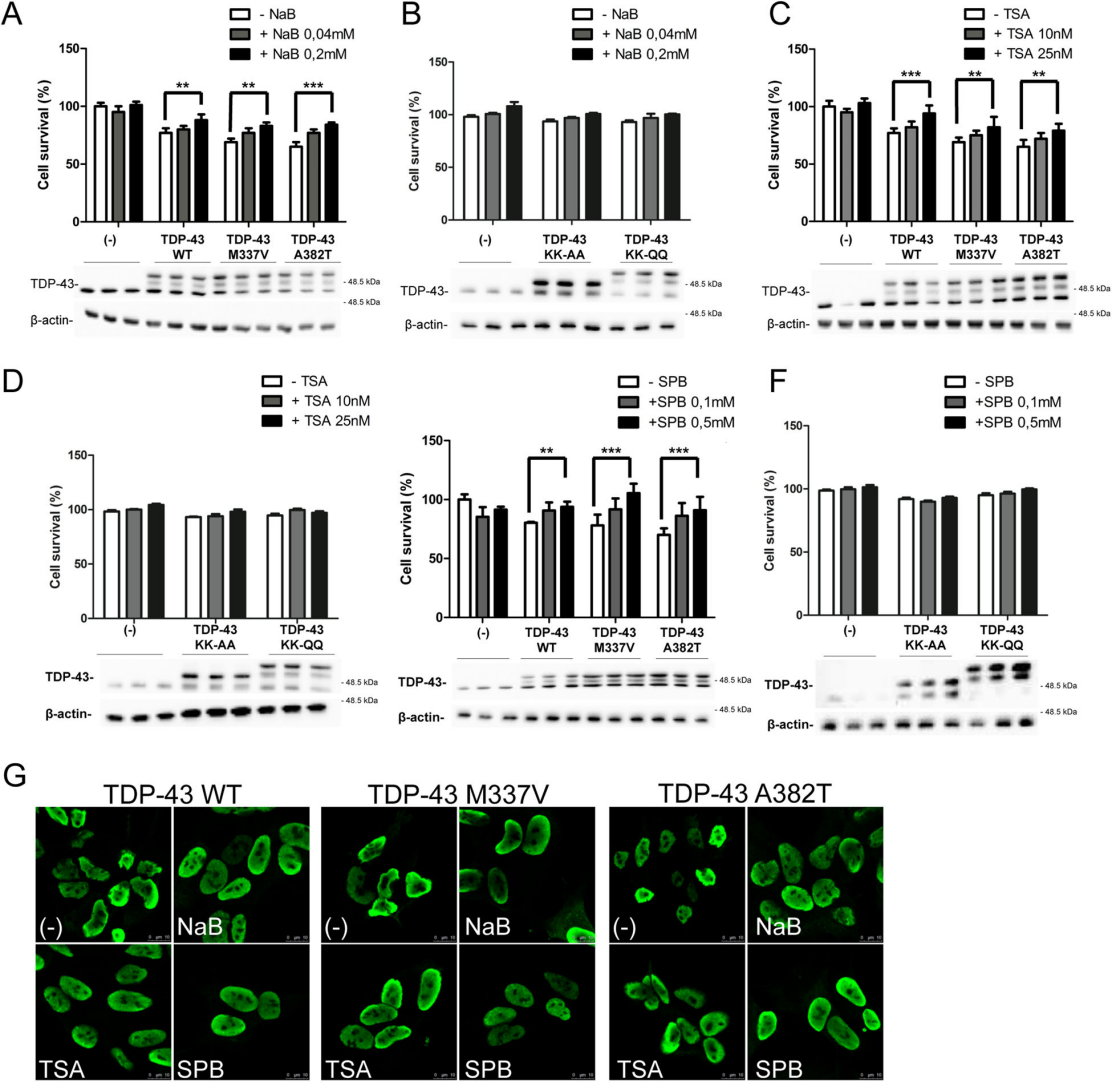
Effect of HDACi on TDP-43-induced cell toxicity in SH-SY5Y cells. MTS assay on SH-SY5Y cells transduced with the indicated adenoviral particles encoding for TDP-43 WT, M337V, A382T, KK-AA, or KK-QQ (MOI 10 pfu/cell), and treated whit different HDACi. Forty-eight hours after transduction and concomitant HDACi treatment cell viability was assessed by a colorimetric assay. At the end of the assay the cell extracts of the four replicates for each time point were pooled and analysed by western blot using anti-TDP-43 antibody. anti-βactin was used as loading control. NaB 0.04 and 0.2 mM (**a**, **b**); TSA 10 nM and 25 nM (**c**, **d**); SPB 0,1 mM and 0,5 mM (**e**, **f**). The data were obtained from four independent experiments; **p > 0.01 and ***p > 0.001 analysed by using one-way ANOVA with Bonferroni’s Multiple comparison post-hoc test (**g**) Immunofluorescence on SH-SY5Y transduced with different myc-tagged-TDP-43 isoforms treated for 48 h with NaB 0.2 mM, TSA 25 nM, and SPB 0.5 mM. Cells were labeled with an anti-Myc antibody and detected with a secondary conjugate to Alexa 488 anti-mouse fluorophore as a secondary antibody. The slides were analysed by Leica confocal microscope. Scale bars = 10 μm.

First, cellular toxicity of the different HDACis was evaluated by testing their dose-response effect on cell viability (Fig. S5) and two different nontoxic concentrations for each inhibitor to be used were defined. Afterwards, SH-SY5Y cells were transduced with recombinant adenovirus coding for WT or different mutants of TDP-43 (M337V, A382T, K145A-K192A, or K145Q-K192Q) and treated by HDACis. These experiments demonstrate an HDACi dose dependent increase in cell survival (Fig. 4). The positive effect of HDAC inhibition on TDP-43-induced cell toxicity was also confirmed in immunofluorescence experiments (Fig. 4g). HDCAi-treated cells display a diffuse staining of TDP-43 in the nuclei, which appear more spherical in respect to the ones of untreated cells. The expression of KK-AA and KK-QQ TDP-43 mutants is less toxic respect to WT TDP-43 and the treatment with HDACis induces a slight increase in cell viability, which does not reach statistical significance.

**Fig. 5:**
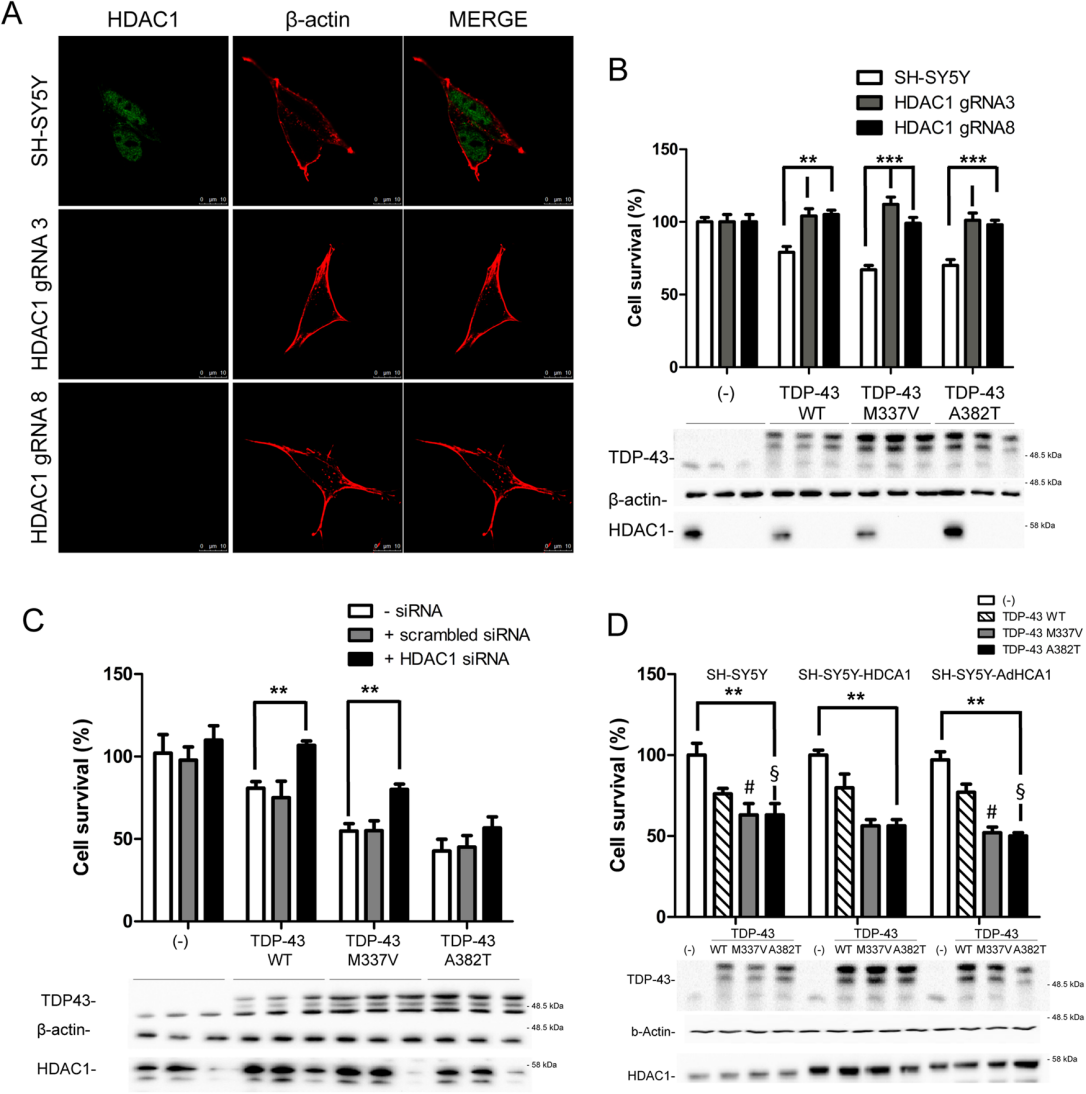
HDAC1 expression level modulates TDP-43-induced cell toxicity. **a** Stable CRISP-Cas9 HDAC1-KO clones were analysed to evaluate HDAC1 expression by immunofluorescence, using anti-HDAC1 and anti-β-actin antibodies. Scale bars = 10μm. **b** MTS assay on two SH-SY5Y-KO cell clones transduced with the indicated adenoviral particles encoding for TDP-43 WT, M337V, A382T. SHSY-5Y cells were used as control. The data were obtained from four independent experiments; **p > 0.01 and ***p > 0.001 versus SH-SY5Y, analysed by using one-way ANOVA with Bonferroni’s Multiple comparison post-hoc test. **c** Cell viability of SH-SY5Y cells treated with HDAC1 or scrambled siRNA, transduced with adenoviruses encoding for TDP-43 WT, M337V, A382T, was calculated after 48 h through an MTS assay. **p > 0.01 versus untreated with HDAC1 siRNA, by using one-way ANOVA with Bonferroni’s Multiple comparison post-hoc test. **d** TDP-43 and HDAC1 have a synergic effect in promoting cell toxicity. SHSY-5Y (white bars), SHSY-5Y-HDAC1 (gray bars, stable cell line expressing HDAC1), and SHSY-5Y, transduced with AdHDAC1 (black bars), were transduced with adenoviral particle coding for WT or pathological mutants (M337V or A382T) TDP-43. Cell viability assay was performed as described in **b**, **c**. **p > 0.01 versus SHSY5Y cells, by using one-way ANOVA with Bonferroni’s Multiple comparison post-hoc test. The two-tailed *P*-value obtained by comparing SHSY5Y cells vs. SHSY5Y-AdHDAC1 expressing TDP-43-M337 or TDP-43 A382T equals, respectively, to 0.075 (#) and 0.0365 (§). Western blotting using anti-TDP-43, anti-HDAC1 and anti-β-actin was performed after MTS assay as described in Fig. 4.

Then HDAC1 expression level was manipulated to assess its effect on TDP-43 toxicity. The CRISPR/Cas9 genome editing technique was used in SH-SY5Y cells to generate stable cell lines in which the expression of HDAC1 was ablated. SH-SY5Y were transfected with a plasmid coding for the humanized Cas9 and different gRNA targeting the second exon of HDAC1 gene. After puromycin selection, single stable clones were isolated and analysed for HDAC1 expression leading to the identification of two lines in which HDAC1 protein expression was absent as demonstrated by immunofluorescence experiment (Fig. 5a) and sequencing (data not shown).

These HDAC1-KO lines were used in cellular vitality tests, after being transduced with TDP-43 expressing adenoviruses. Interestingly, the ablation of HDAC1 expression significantly ameliorates TDP-43-mediated cell death (Fig. 5b).

To confirm the evidence coming from HDCA1 genetic ablation, a commercial HDAC1 small interfering RNA (siRNA) to downregulate HDAC1 was used. Thus, we observed that the reduction of HDAC1 protein level by 70%, which causes a statistically significant decrease of TDP-43-induced cell toxicity, compared with the random sequence control (Fig. 5c).

Conversely, TDP-43 toxicity was exacerbated (Fig. 5d) when HDAC1 was transiently overexpressed via adenoviral transduction (Fig. S7) and at a less extent in SH-SY5Y stably expressing HDAC1 (Fig. S6).

### Expression of human TDP-43 in fly eyes leads to progressive eye degeneration partially suppressed by HDAC1 silencing

The present study demonstrated that in three different experimental paradigms (genomic inactivation, siRNA, and HDACi treatment) the reduction of HDAC1 activity significantly reduces TDP-43-mediated cell death. To confirm these results through an in vivo approach, Drosophila ALS models^37^ were exploited.

**Fig. 6:**
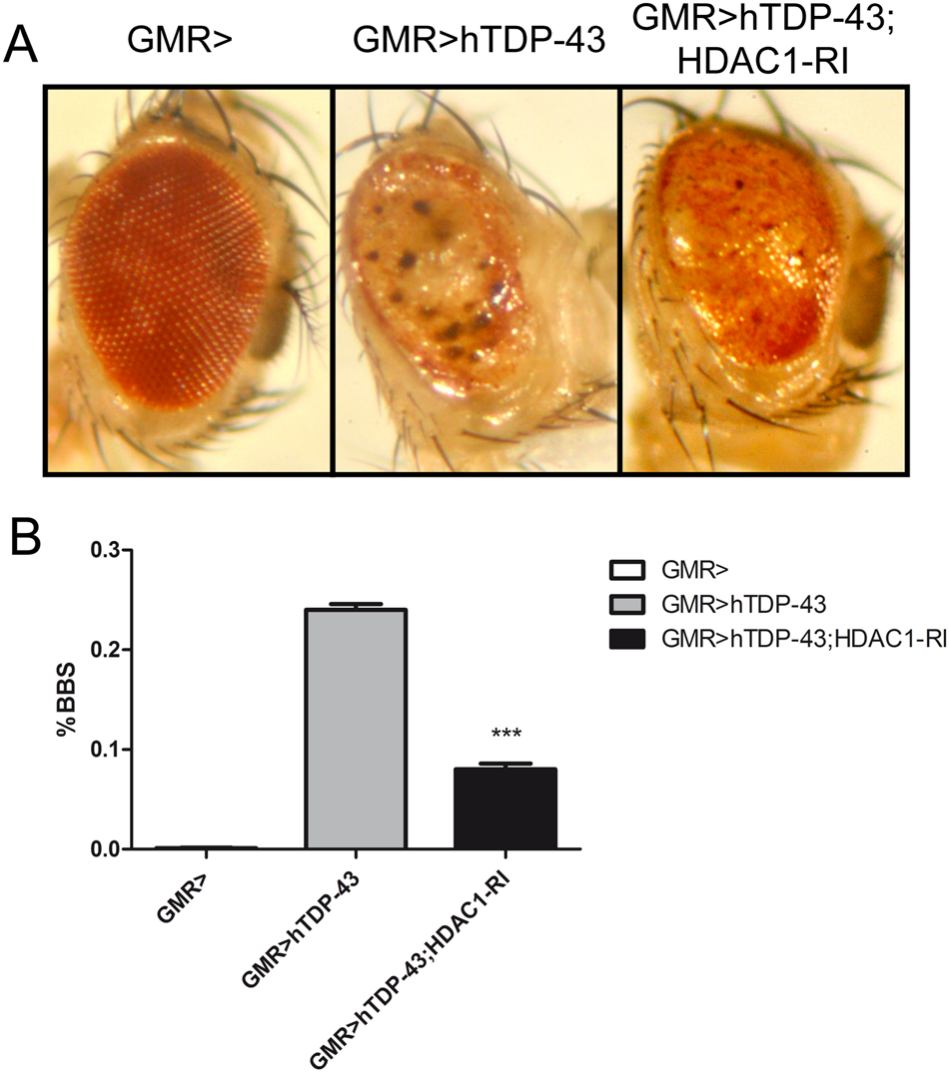
HDAC1 silencing reduces cell death and neurodegeneration induced by hTDP-43 expression in Drosophila eye. Eyes of flies expressing different transgenes under control of GMR-Gal4, are shown (**a**). TDP-43 on its own induces strong eye degeneration and the appearance of dark spots (BBS big black spots), which are an indication of massive cell death. RNAi-mediated downregulation of dHDAC1 significantly improves TDP-43-mediated neurodegeneration and cell death, as indicted by the reduction of dark areas of degeneration. **b** Graphic representation of the data (**a**), in which we counted the number of eyes showing cell death areas (BBS) in fly expressing TDP-43 on its own, or together with dHDAC1 RNAi construct, and we compared their frequencies. Statistical significance was tested by CHI-square (Yates correction).

As shown in Fig. 6, expression of human TDP-43 in drosophila eye leads to a well described retinal degeneration^45^, which is associated to a strong cell death phenotype characterized by depigmentation, roughness, and dark spots. When these flies were crossed with a line in which Rpd3, the HDAC1 and HDAC2 drosophila ortholog, is inactivated by the expression of an RNAi specific construct^46,47^, a reduction in retinal degeneration can be clearly observed. These eyes exhibit a significant reduction of the dark apoptotic areas (Fig. 6).

These data strongly demonstrate that HDAC1 silencing in Drosophila, as well in SH-SY5Y cells, is able to ameliorate the toxic effect induced by TDP-43 expression.

## Discussion

By using cellular and animal models we were able to describe a specific interaction between HDAC1 and TDP-43, via RRM1 and RRM2 domains. Interestingly, our experimental results suggest that HDAC1 may play a pivotal role on the deacetylation of TDP-43 in the nucleus. In addition, we observed as the impairment of TDP-43 deacetylation, by TDP-43 mutagenesis or by genetic/pharmacological HDAC1 inhibition, has a positive effect on TDP-43-induced cell death. Notably, through ChIP analysis and luciferase assays, evidence is provided that TDP-43 is a direct activator of CHOP (C/EBP‐homologous protein) transcription, expanding what previously reported^32^. CHOP is a mediator of cell death, caused by the activation of the unfolded protein response, a key event in the ALS-linked proteinophaties^48^. TDP-43 acetylation-mimicking mutant (TDP-43 QQ) loses its ability to induce CHOP transcription, indicating that TDP-43 acetylation can affect TDP-43-mediated pathways downstream ER stress, as well as further promote the nuclear retention of TDP-43 upon exposition to stressful signals.

The phenotype described is in line with the multiple crossroads between epigenome, epigenetic machinery, and ALS, described in the past 10 years^14,49^.

We had previously demonstrated that TDP-43 M337V expression induces a decrease in global histone H3 phosho-acetylation^33^, that can affect TDP-43-induced cell toxicity at multiple levels, since changes in acetylation of both histone and nonhistone proteins have been reported to affect cell physiology spanning from transcription, to DNA repair signal transduction, and protein aggregation^50^.

HDACs are key modulators of the acetyloma and they have been demonstrated to be deregulated in ALS experimental models and patients^13^. In fact, the levels of HDAC1, HDAC2, and sirtuins (a family of class III HDACs) are impaired in post-mortem ALS tissues^17^. In yeast Set-3, a component of the histone deacetylation complex, is a modulator of TDP-43 toxicity^51^. HDAC1, like most HDCAs, is a nuclear enzyme that can be re-localized in the cytosol in damaged axons of demyelinating models, such as in patients affected by multiple sclerosis and in cultured neurons exposed to glutamate or TNF-α^52,53^. More recently, during development of the Xenopus brain, HDAC1 was observed in the mitochondria of developing neurons^54^. In addition, in a FUS knock-in mouse model HDAC1 is mislocalized to the cytoplasm^55^, probably following dephosphorylation on serine 421 and 423^53^. Despite this evidence we were not able to highlight HDAC1 re-localization in our cellular models.

HDACis were originally applied to cancer therapy and some of them, such as panobinostat, have been approved from FDA for multiple myeloma treatment, while others, like VPA and SPB, are in phase II or III clinical trials, respectively, for hematological and solid malignancies^40^. At present more than 350 clinical trials involving HDACi have been carried out or are on-going not only as single therapeutic but also in combination with other targeted agents against various human diseases, including neurodegenerative diseases. VPA was approved by FDA in 1978 as an anticonvulsant drug for the treatment of seizure disorders, even if the molecular target of this drug it is not known yet. The possibility of using HDACi for neurodegenerative treatment originates in 2008, when Hahnen identified two major HDACi neuroprotective mechanisms, including the transcriptional activation of disease-modifying genes and the rectification of destabilization in histone acetylation homeostasis^56^. Several pan-HDACis reduced ALS development in mice^43,57,58^. SPB was shown to extend survival and motor performance in transgenic ALS SOD1 animal model, and these effects were attributed to an upregulation in the expression of nuclear factor *κ*B (NF-*κ*B) and bcl-2 proteins^41^, although the genetic inhibition of NF-*κ*B in SOD1 mice does not ameliorate disease onset and progression^59^. Unfortunately, even if VPA and SPB are safe, tolerable, and efficient in improving histone acetylation levels, they failed to ameliorate clinical parameters in ALS patients^42,60^. In line with this observation the work by Pigna et al. identified HDAC4, a class IIa HDAC, as having a crucial role in preserving the innervations and skeletal muscle in SOD1 ALS mouse model^61^. HDAC4 genetic ablation in skeletal muscle accelerates ALS pathological features, indicating a possible risk for using HDACs pan-inhibitors in ALS treatment. On the other hand, HDAC6 inhibition in motor neuron cultures derived from iPSCs, originated from fibroblasts of ALS patients carrying different *FUS* mutations, reverses axonal transport defects^62^. Treatment of FUS transgenic mice with ACY-738, a potent class I HDAC brain penetrable inhibitor, largely restores global histone acetylation, and metabolic gene expression in the spinal cord^63^. ACY-738 inhibits HDAC6 with low nanomolar potency and a selectivity of 60- to 1500-fold over class I HDACs, but its effect in FUS mouse model is independent from HDAC6 itself, indicating that other members of the family, including HDAC1, can be the key element mediating the observed therapeutic effects^63^. Moreover HDAC1 appears as a downstream target of both FUS and TDP-43 related ALS in mediating double strand-breaks repair^20,22,23,64,65^.

Although HDACi translational failure underlies ALS complexity and can be related to the lack of selectivity for different HDACs, more specific drugs would be very useful. Particularly, evidence provided indicates that HDAC1 inhibition can be a precious therapeutic option in ALS therapy.

## Material and methods

### Antibodies and reagents

The following primary antibodies were used in this study: Myc monoclonal antibody (M4439, Sigma-Aldrich), β-actin (A5441, Sigma-Aldrich), Flag (F3165, Sigma-Aldrich), HDAC1 (10197-1-AP, Proteintech), TARDBP (190782-2-AP, Proteintech), Acetylated-Lysine antibody (9441, Cell Signaling), GFP (33-260, ThermoFisher Scientific), caspase-3 (♯9662, Cell Signaling Technology), PARP (♯9542, Cell Signaling Technology), LC3B (♯2775, 2Cell Signaling Technology), anti-rabbit peroxidase-conjugated secondary antibody (AP132P EMD Millipore) and anti-mouse peroxidase-conjugated secondary antibody (AP124P EMD Millipore); anti-rabbit, anti-mouse Alexa 488 (A-11001, Life Technologies) or 647-conjugated secondary antibody (A-21244, Life Technologies). All antibodies were used at the dilution recommended by the manufacturer’s instructions.

The following HDACis were used in this study: Sodium phenil butyrate (SML0309, Sigma-Aldrich), Trichostatin A (T8552, Sigma-Aldrich), Sodium butyrate (B5887, Sigma-Aldrich), Valproic acid sodium salt (P4543, Sigma-Aldrich).

### Mice tissue

Mice tissues were dissected from BALB31c mice housed at the *Istituto Zooprofilattico della Sardegna (Sassari, Italy)*. All animal procedures have been performed according to the European Guidelines for the use of animals in research (86/609/CEE) and the requirements of Italian laws (D.L. 116/92, Directive 2010/63/EU). The ethical procedure has been approved by the Animal welfare office, Department of Public Health and Veterinary, Nutrition and Food Safety, General Management of Animal Care and Veterinary Drugs of the Italian Ministry of Health (Application number 32/08 of 7 July 2008; Approval number 744 of 9 January 2009). Authorized investigators performed all the experiments. Dissected tissue was immediately frozen in liquid nitrogen and stored at −80 °C.

### Drosophila model

Flies expressing insect codon-optimized version of human wild-type TDP-43 (gl-TDP-43CO)^66^ under UAS promoter during fly eye development, driven by GMR-Gal4, were crossed at 25 °C with flies expressing a unique dsRNA that targets Drosophila HDAC1. In particular, GMR-GAL4 on X-chromosome was originally placed in trans to generate a GMR-GAL4;gl-TDP-165 43CO/CyO transgenic fly, which was obtained from Bloomington Stock Center^67^. RNAi line to target HDAC1 (v30599) (also named Rpd3 in Drosophila), which was previously utilized to downregulated Rpd3 according to^46,47^, was obtained by Vienna Drosophila Research Center. Eye neurodegeneration was evaluated according to^68^. A minimum of 60 flies were randomly choosed, with investigators blind to the genotypes, during the analysis.

### Plasmids construction and oligonucleotides

Sequence coding for human TDP-43 (NM_007375.3) or human HDAC1 (NM_004964.2) were cloned in different expression vectors (pCS2-MTK, pCMV-3xFlag or pShuttle2) and used for site-directed mutagenesis (QuickChange site-directed mutagenesis kit, Agilent). Mutants were obtained by mutagenesis starting from hTDP-43, by site-directed mutagenesis. Positive clones were screened by sequencing. Human HDAC6 (NC_000023.11) and HDAC2 (NM_001527) were cloned in expression vector pCMV-3xFlag.

### Adenoviral particle production

All adenoviral vectors (*pAdenoX-hTDP-43WT/Q331K/M337V/A382T/ΔRRM1-2/K145A-K192A/K145Q-K192Q and pAdenoX-hHDAC1*) were generated using the Adeno-X Expression System 1 (Clontech) and partially described. All constructions were verified by automated sequencing. Adenoviral particles were produced and titrated using the Adenoviral-X Expression System 1 (Clontech) according to manufacturer’s instruction. Cells were transduced by adenoviral particles (5–30 pfu/cell) in DMEM–F12 and incubated at 37°C for 1 h. The transduced cells (usually more than 90% expressing TDP-43) were analysed 48 h transduction.

### Cell lines and culture

Human neuroblastoma SH-SY5Y cells (CRL-2266, ATCC, Rockville, MD) and SH-SY5Y-CFP-DEVD-YFP^38^ cells were grown in DMEM–F12, 10% Fetal Bovine Serum (FBS) at 37 °C. The plasmid pcDNA3 containing cDNA coding for 3 × Flag-HDAC1 was transfected using Lipofectamine^®^ LTX Reagent (Life Technologies) according to the manufacturer’s protocol. The different SH-SY5Y clones were maintained under selection by 400 μg/mL of G418. Individual clones were picked after 14 days of selection, moved in a 96 well plate, and maintained under selective medium until confluence growth. Different individual clones were analysed for HDAC1 expression by western blot and immunofluorescence.

To expose cells to stressful conditions after transduction with the indicated adenoviral particle, cells were either exposed to NaArs 50 µM for 16 h or for UV irradiation: cells were treated with UV-C (254 nm) using a low pressure mercury lamp, and the cells were subjected to global (2.5 J/m^2^). After microirradiation, cells were incubated for 4 h at 37 °C in a humidified atmosphere containing 5% CO_2_. All slides were processed by assaying the previously mentioned immunofluorescence protocol and analysed by confocal microscopy.

#### Adeno-X 293 cell line

Adenovirus 5-transformed Human Embryonic Kidney 293 cell line (CRL 1573 HEK 293; ATCC, Rockville, MD,) was used to package and propagate the recombinant adenoviral- based vectors produced with the BD Adeno-X Expression System.

SH-SY5Y cells (ATCC number CRL-2266) were grown in DMEM–F12, 10% FBS at 37 °C whereas, *Adeno-X 293 cell line* were grown in DMEM, 10% FBS at 37 °C. Transient expression of each vector (2,5 µg DNA/1 × 10^6^ cells) was obtained with Lipofectamine Plus reagent (ThermoFisher) according to manufacturer’s instructions. After an incubation of 4 h with transfection reagents, the cells were cultured in normal growth medium for 24 or 48 h. Trasduction with adenoviral particle with a MOI of 5–10 pfu/cell was performed according to^69^).

### Co-immunoprecipitation

Briefly, cultured cells were lysed with lysis buffer (120 mM NaCl, 50 mM Tris pH 7.5, 5 mM EDTA, 0.5% NP-40, and 1 mM freshly prepared PMSF), containing protease inhibitors (SIGMA P 8340). Cell lysates were immunoprecipitated overnight at 4 °C with specific antibodies; immunocomplexes were then captured by incubating for 16 h at 4 °C with continuous gentle shaking, with protein-A sepharose from *Staphylococcus aureus* (Sigma-Aldrich P3391). Subsequently, immunocomplexes were analysed by means of western blotting, using specific antibodies.

### SDS-PAGE and western immunoblotting

Protein content was determined using Bradford protein assay (27813 SIGMA). Equal amounts of protein extracts were resolved by standard SDS/PAGE. Samples were then electroblotted onto Protan nitrocellulose membranes (GE Healthcare Life Science). Afterwards, membranes were incubated in 3% low-fat milk, diluted in 1 × PBS-Tween 0.05% solution with the indicated antibody for 16 h at 4 °C. Anti-Rabbit IgG (whole molecule)- and Anti-Mouse IgG (whole molecule)-peroxidase antibody (EMD Millipore) were used to reveal immunocomplexes by enhanced chemioluminescence (ThermoFischer). The apparent molecular weight of proteins was determined by calibrating the blots with prestained molecular weight markers (Bio-Rad, Hercules, CA). Where indicated, the relative signal intensity acquired by using the ChemiDoc XRS+ (Bio-Rad, Hercules, CA) was quantified using QuantityOne Software.

### Two-dimensional electrophoresis analysis

Two-dimensional electrophoresis (2-DE) was used to separate proteins according to their isoelectric point (1st dimension) and, orthogonally, to their molecular weight (2nd dimension).

2-DE was performed as reported elsewhere^70,71^. Briefly, samples were applied to 70 mm IPG strips (pH 4-7, Bio-Rad, Hercules, CA), by overnight rehydration loading at 20 °C, and then isoelectrofocused at 50 μA/IPG strip for 22 kVh at 20 °C. Once isoelectric focusing was completed, proteins were in-gel reduced by incubating IPG strips with 50 mM tris buffer containing 6 M urea, 30% glycerol v/v, 3% SDS w/v, and 1% DTT w/v, followed by in-gel alkylation with the same solution containing 2.5% iodoacetamide w/v, in place of DTT. Each step was performed keeping strips under continuous shaking for 15 min. IPG strips were then sealed with 0.5% low melting point agarose w/v, in SDS running buffer, at top of second dimension gels (8 × 7 cm × 0.1 cm). SDS-PAGE was carried out using 15% T, 3% C polyacrylamide gels at the following conditions: 50 V for 15 min and subsequently at 150 V until the Bromophenol dye front reached the lower limit of the gel, in a Mini-Protean Tetra Cell (Bio-Rad, Hercules, CA). Later gels were subjected to western blot analysis, as described above.

### Immunofluorescence

Cells were grown on a cover-glass, were washed twice with PBS 1X and then fixed with 4% paraformaldehyde in 1 × PBS and permeabilized with 0.2% Triton X-100 in 1 × PBS. After a blocking step for 1 h in 5% BSA, diluted in 1 × PBS-0.05% Tween-20, cells were incubated with the primary antibody mouse anti-Myc (Sigma-Aldrich), diluted 1∶10000 in blocking solution, overnight at 4 °C, and then incubated with a secondary antibody Alexa Fluor^®^488 goat anti-mouse IgG (Life Technologies), diluted 1∶1000 in blocking solution, for 1 h at the room temperature. Cells were then analysed with a Leica TCS SP5 confocal microscopy, with LAS lite 170 image software.

### Chromatin immunoprecipitation (ChIP)

SH-SY5Y cells (4 × 10^6^) were plated 24 h before transduction, and infected by using viruses encoding for TDP WT at a multiplicity of 30 pfu/cell. After 24 h, cells were harvested and chromatin immunoprecipitation was performed using EZ-Magna ChIP™ (Millipore), according to the manufacturer’s protocol.

Each immunoprecipitated (IP) reaction was performed using about 1 × 10^6^ cells equivalents of chromatin. The antibodies used for immunoprecipitation were the following: TARDBP Polyclonal Antibody (Proteintech_10782-2-AP) and Normal Rabbit Ig (reagent supplied) as negative control. Purified chromatin was eluted and DNA fragments were used for qPCR (S2). The results were normalized using the Fold Enrichment Method (ChIP signals were divided by the no-antibody signals, representing the ChIP signal as the fold increase in signal relative to the background signal). **p < 0,01 Student’s *t* test.

### Luciferase activity assay

The DNA-damage-inducible transcript 3 (gene-synonym CEBPZ, CHOP, GADD153^72^) promoter from −954 to +91 was cloned between the *XhoI* and *HindIII* sites in the pGLE-Basic Vector. All constructions were verified by automated sequencing. SH-SY5Y cells were seeded in 24-well plates and cultured for 16 h. Cells were then transfected by wild-type or mutant of TDP-43, HDAC1 and luciferase constructs, in addition to a Renilla vector, used as an internal control for luciferase activity; transfected cells were further cultured for 48 h. Luciferase assays were conducted using dual luciferase assay system (Promega). Each experiment was performed in triplicate.

### siRNA on HDAC1 mRNA

SH-SY5Y cells (1 × 10^5^) were seeded 24 h before the first transfection with the siRNA oligonucleotide specific for HDAC1 gene. Lipofectamine 3000 reagent (Lipofectamine^®^ 3000, ThermoFisher) was combined with Optimem medium (Promega) (reaction 1); meanwhile, in a different tube, 148 pmol of HDAC1 siRNA and 500 ng of TDP, WT or mutant, were mixed with Optimem (reaction 2). Both reactions were mixed and left for 5 min at the room temperature. Afterwards, the mixture was added to the cells and incubated at 37 °C and 5% of CO_2_. After 48 h the transfection procedure was repeated only for HDAC1 siRNA, and cells were incubated at 37 °C and 5% of CO_2_ for other 24 h. After 72 h a MTS assay was performed, and cells were finally washed two times with PBS; subsequently, cell lysates were subjected to western blot analysis with anti-HDAC1 antibodies to evaluate expression level.

### Design of targeting components and the use of the CRISPR Design Tool

The web interface of CRISPR Design Tool (http://tools.genome-engineering.org) was used to develop gRNAs. Off-target activity was evaluated additionally with Blastn (https://blast.ncbi.nlm.nih.gov/Blast.cgi). The pSpCas9(BB)-2A-Puro (Addgene # 48139) that expresses the Streptococcus pyogenes Cas9 (including an NLS and a FLAG tag) from a CAG promoter and has a U6 promoter driven gRNA, was used as cloning backbone according to^73^. Briefly, phosphorylation and annealing were performed with the three pairs of oligos, mentioned above, harboring a BbsI overhang. Afterwards, BbsI (#FD1014, ThermoFisher) mediated digestion and T4 DNA ligase (#M0318L, NEB) directed ligation in the linearized pSpCas9(BB)-2A-Puro was performed. After the transformation, cloning was verified with a control PCR with the primers in the table. Plasmids were purified and sequenced. After transfection of the indicated combinations of pSpCas9(BB)-2A-Puro-gRNAs (Addgene #62988)), positive cells were selected using puromycin (2 μg mL^−1^) for 5 days prior to clonal expansion. Empty pSpCas9(BB)-2A-Puro was used as negative control.

### MTS assay

Cell viability was assessed by a colorimetric assay using 3(4,5-dimethylthiazol-2yl)-5-(3-carboxymethoxyphenyl)-2-(4- sulfophenyl)-2H-tetrazolium (MTS) assay (Cell Titer 96 Aqueous One Solution Assay, Promega), according to manufacturer’s instructions. Absorbance at 490 nm was measured in a multilabel counter (Victor X5, PerkinElmer) 72 h post trasnduction.

### Statistical analysis

The results are presented as means ± S.D. of *n* ≥ 3 independent experiments. Statistical evaluation was conducted by one-way or two-way ANOVA and Bonferroni post test. Values significantly different from the relative control are indicated with a symbol: *p < 0,05; **p < 0.01; ***p < 0.001 or ^§^*p* < 0.05; ^§§^*p* < 0,01; ^§§§^*p* < 0.001.

## Supplementary information

Supplementary Figure legends-clean copy

Figure S1-rev

Figure S2

Figure S3

Figure S4-rev

Figure S5

Figure S6-rev

Figure S7-rev
