# Supplementary material for: HDAC1 inhibition ameliorates TDP-43-induced cell death in vitro and in vivo
Source: Cell Death Dis. 2020 May 14;11(5):369. doi: 10.1038/s41419-020-2580-3 (PMC7224392; doi:10.1038/s41419-020-2580-3)
Supplement: Supplementary file 1 — Supplementary Figure legends-clean copy [file 41419_2020_2580_MOESM1_ESM.docx]

**Supplementary figures**

Primer for site-directed mutagenesis

| Mutation | Oligonucleotides |
| --- | --- |
| M337V | 5’CAGTTGGGGTATGGTGGGCATGTTAGC 3’ |
| A382T | 5’AATTCTGGTGCAACAATTGGTTGGG3 3’ |
| K145A-K192A | 5’ GATCTTAAGACTGGTCATTCAGCGGGGTTTGGCTTTGTTCG 3’ |
| K145Q-K192Q | 5’ GCCTTTGAGAAGCAGACAAGTGTTTGTGGGGCGCTGTACAG 3’ |
| K236E | 5’ TCCAATGCCGAACCTGAGCACAATAGCAATAGA 3’ |
| D169G | 5’ TTCTCTCAGTAGGGGGTGTGAT 3’ |
| ΔNterm | 5’ GAATTCAAGGCCTCTCGAGCCATAGTGTTGGGTCTCCCATGG 3’ |
| ΔRRM1 | 5’ CAGAAAACATCCGATTTAATAGTGCCTAATTCTAAGCAAAGC 3’ |
| ΔRRM2 | 5’ GAGCCTTTGAGAAGCAGAAAACACAATAGCAATAGACAG 3’ |
| ΔG-rich | 5’ CAGTTAGAAAGAAGTGGAAGAGGAATGTAGCTCGAG 3’ |
| ΔRRM1- ΔRRM2 | 5’ CAGAAAACATCCGATTTAATAGTGCCTAATTCTAAGCAAAGC 3’ |
| gRNA1 HDAC1F | caccgTGAGTCATGCGGATTCGGTG |
| gRNA1 HDAC1R | aaacCACCGAATCCGCATGACTCAC |
| gRNA3 HDAC1F | caccgGATACCAGAGATGGCTTTTT |
| gRNA3 HDAC1R | aaacAAAAAGCCATCTCTGGTATCC |
| gRNA6 HDAC1F | caccgCCCAATGAAGCCTCACCGAA |
| gRNA6 HDAC1R | aaacTTCGGTGAGGCTTCATTGGGc |

Primers for creating the HDAC1-KO using the CRISP/Cas9 system

| gRNA1 HDAC1F | caccgTGAGTCATGCGGATTCGGTG |
| --- | --- |
| gRNA1 HDAC1R | aaacCACCGAATCCGCATGACTCAC |
| gRNA3 HDAC1F | caccgGATACCAGAGATGGCTTTTT |
| gRNA3 HDAC1R | aaacAAAAAGCCATCTCTGGTATCC |
| gRNA6 HDAC1F | caccgCCCAATGAAGCCTCACCGAA |
| gRNA6 HDAC1R | aaacTTCGGTGAGGCTTCATTGGGc |

**Table 1. Sequenze of the primer used**

**Legends to supplementary figures**

**Figure S1**. (**A**) Immunofluorescence analysis of WT or mutants TDP-43. SH-SY5Y cells were transfected with the indicated plasmid and 48h after cells were fixed and incubated with anti-myc antibody to detect TDP-43. The slides were analyzed by Leica confocal microscope. Scale bars = 10μm. (**B**) HEK 293T cells were transiently transfected with FLAG-tagged HDCA1 or Flag-tagged-HDAC2 and Myc-tagged TDP-43. 48h after transfection, cells were lysed, protein extracts were immunoprecipitated and proteins retained were separated on SDS-PAGE and visualized by western blot using specific antibodies.

**Figure S2**. **Immunofluorescence analysis of HDAC1 localization upon WT or mutants TDP43 expression.** SH-SY5Y cells were transfected with the indicated plasmid and 48h after cells were fixed and incubated with anti-Myc antibody to detect TDP-43 and anti-HDAC1. The slides were analyzed by Leica confocal microscope. Scale bars = 10μm

**Figure S3. Schematic representation of CHOP promoter.** The primer used in ChIP analysis are indicated in (**A**) and their sequence in (**B**).

**Figure S4. Effects of adenoviral-mediated expression of WT or mutant TDP-43 on cell viability**. **A**) MTS assay on SH-SY5Y cells transduced by adenoviral particles coding for TDP-43 WT, M337V, A382T, ΔRRM1-2, KK-AA and KK-QQ, with a MOI of of 5, 10, 20 and 40 pfu/cell, and analyzed 48 hours after transduction. The intensity of each single band was quantified using the QuantityOne software. The data were obtained from four independent experiments; * indicates p< 0.05, ** p> 0.01 and *** p > 0.001 analyzed by using two-way ANOVA with Bonferroni’s Multiple comparison post-hoc test, comparing each column with control cells transduced with a MOI of 5 pfu/cell **B**) Cell lysates were subjected to SDS-PAGE and western blot. The anti-TDP-43 antibody was used to visualize TDP-43 expression level (the symbol **>** points at Myc-tagged-TDP-43) and β-actin serves as controls for equal loading. **C**) Western blot analysis performed on cell lysates from SHSY5Y cells stably expressing the apoptotic sensor myc-CFP-DEVD-YFP (Sanna et al., 2014) transduced with increasing concentration of adenoviral particles coding for TDP-43 as in (A) using anti GFP, anti-caspase-3, anti-TDP-43 and anti- β-actin. Cells trated for 4h with staurosporine (STS) 1μM were used as positive controls for apoptosis. **D**) Western blot analysis performed on cell lysates from SHSY5Y trasnduced as in (A) using anti-PARP and anti-LC3B antibodies.

**Figure S5. HDAC inhibitor Dose-response effects on cell viability in SHSY5Y cells.** SH-SY5Y cells were treated with the indicated HDACi at different doses for 48h. NaB 0,04mM, 0,2mM or 0,1 mM; TSA 10nM, 25nM or 100nM; SPB 0,1mM, 0,5mM or 1mM. The data were obtained from four independent experiments; * indicates p< 0.05 analyzed by using one-way ANOVA with Bonferroni’s Multiple comparison post-hoc test.

**Figure S6. Levels of expression of Flag-tagged-HDAC1 in different stable cell clones**

SHSY5Y cells were transfected with a plasmid coding for HDAC1 fused in frame with FLAG tag. After selection with G418 400 ug/ml three single clones were isolated and tested for HDAC1 expression by (**A**) western blot, using anti-HDAC1 and anti-β-actin antibodies on protein extracts from different clones expressing HDAC1. Evaluation of HDAC1 expression in the cell lines SH-SY5Y-HDCA1-clone 2 by (**B**) confocal microscopy, using monoclonal anti-FLAG and anti-β-actin antibodies and by (**C**) western blot using monoclonal anti-HDAC1, anti-FLAG and anti-β-actin antibodies. Signal intensity was determined using Gel Doc XR software. Intensity data of total HDAC1 (endogenous and flag tagged-HDAC1) and βactin were used to calculate the ratio of HDAC1 overexpression.

**Figure S7. Levels of expression of Flag-tagged-HDAC1 in cells transduced with adenoviral particlesand their effects on cell viability.** **A**) Western blot analysis on protein extracts from SH-SY5Y cells transduced with increasing dose of HDAC1 adenoviruses. 48h after transduction protein extracts were subjected to SDS-PAGE and western blot, with anti-flag, anti HDAC1 or anti β-actin antibodies. **B**) MTS assay on SH-SY5Y cells transduced as in A) at a final concentration of 5, 10, 20 and 40 PFU/cell and analyzed 48 hours after transduction. Intensity data of total HDAC1 (endogenous and transduced flag tagged-HDAC1) and β−actin were used to calculate the ratio of HDAC1 overexpression.The data were obtained from four independent experiments; * indicates p< 0.05 analyzed by using one-way ANOVA with Bonferroni’s Multiple comparison post-hoc test. The anti-HDAC1 antibody was used to visualize HDAC1 expression level and β-actin as controls for equal loading of samples. **C**) Immunofluorescence analysis on cell as in B.

Sanna V, Pala N, Dessì G, Manconi P, Mariani A, Dedola S, Rassu M, Crosio C, Iaccarino C, Sechi M. (2014) "Single step green synthesis and characterization of gold-conjugated polyphenol nanoparticles with antioxidant and biological activities", International Journal of Nanomedicin. 9:4935-51. doi: 10.2147/IJN.S70648. eCollection 2014.
