# Supplementary figures and images for: HDAC1 inhibition ameliorates TDP-43-induced cell death in vitro and in vivo
Source: Cell Death Dis. 2020 May 14;11(5):369. doi: 10.1038/s41419-020-2580-3 (PMC7224392; doi:10.1038/s41419-020-2580-3)

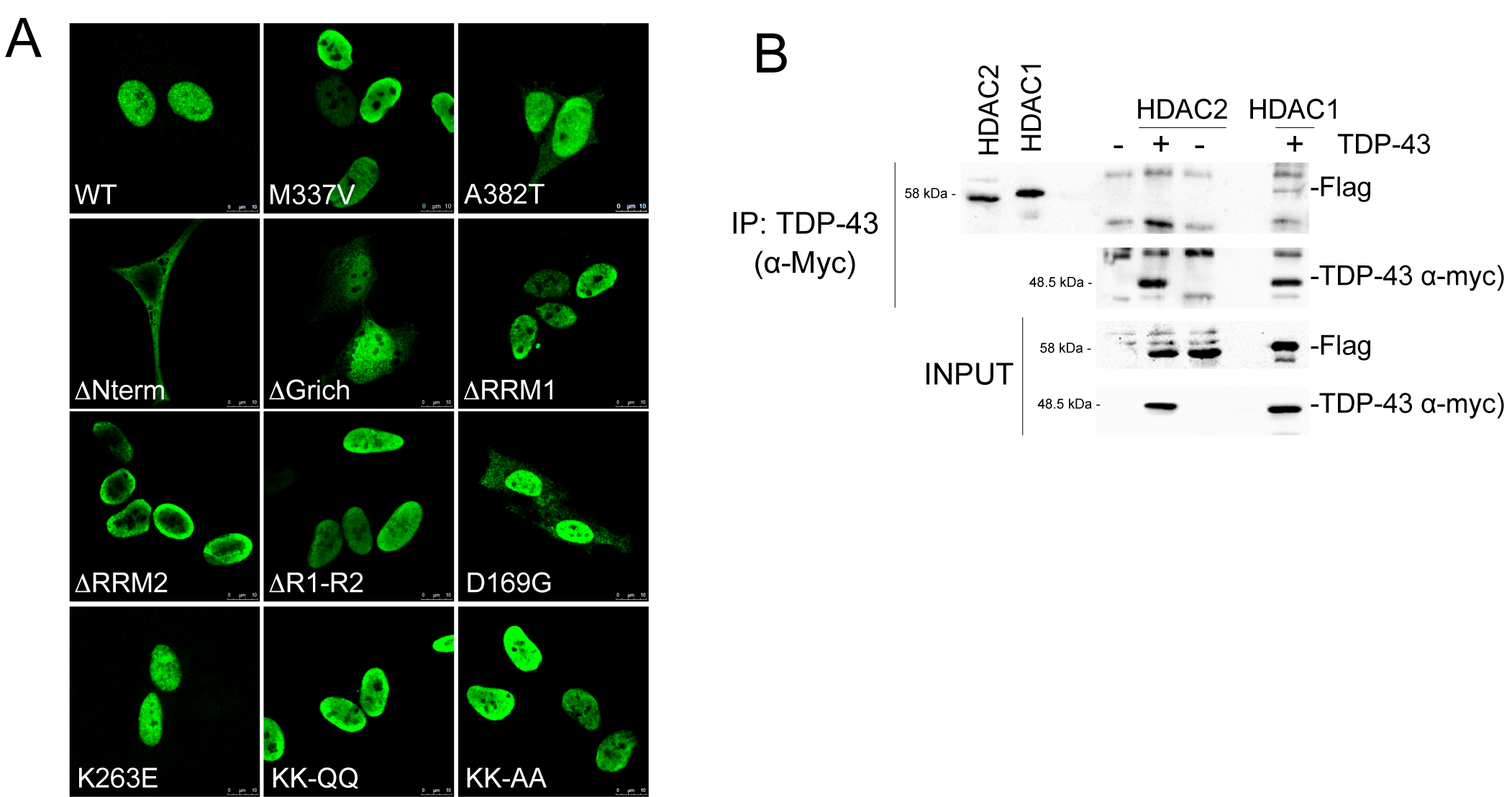

Supplement: Supplementary file 2 — Figure S1-rev [file 41419_2020_2580_MOESM2_ESM.tif]

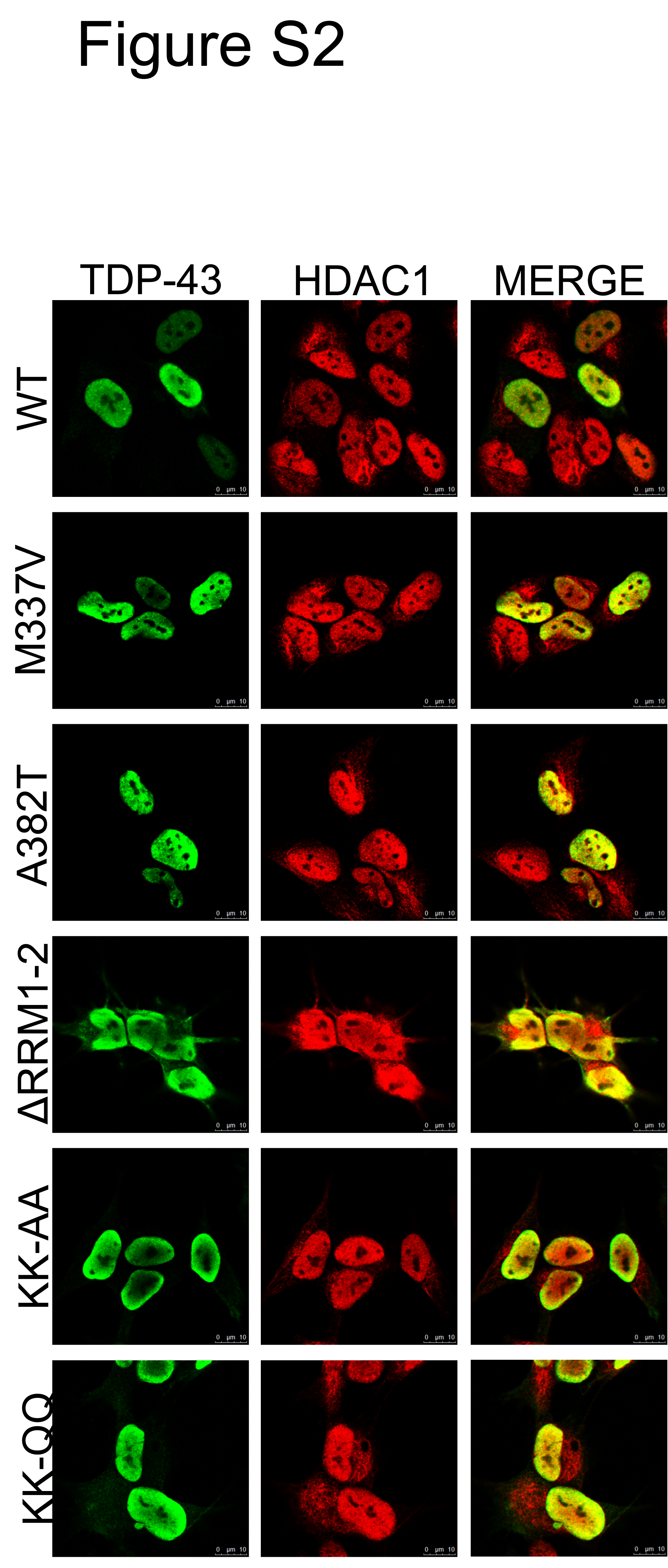

Supplement: Supplementary file 3 — Figure S2 [file 41419_2020_2580_MOESM3_ESM.tif]

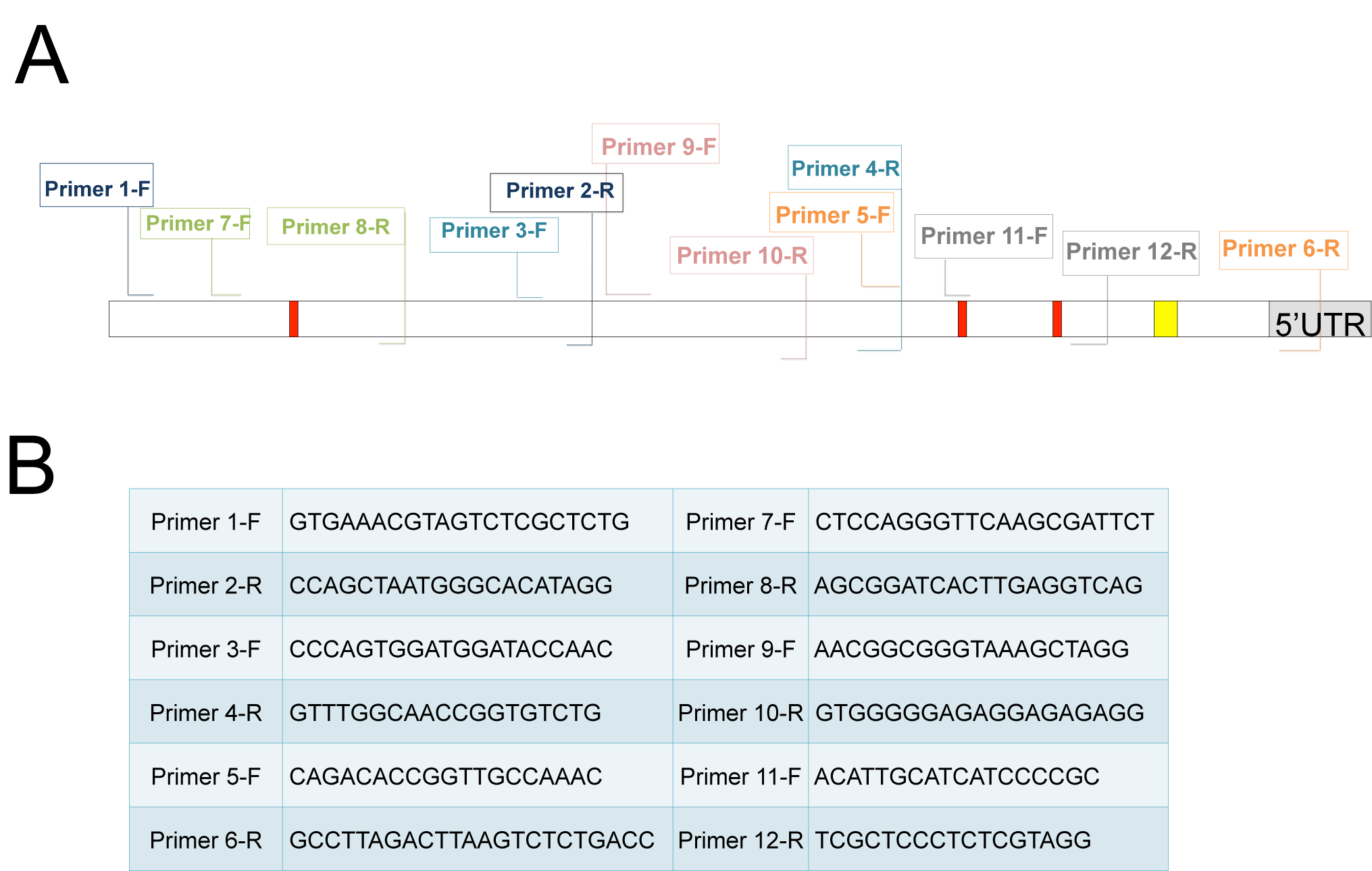

Supplement: Supplementary file 4 — Figure S3 [file 41419_2020_2580_MOESM4_ESM.tif]

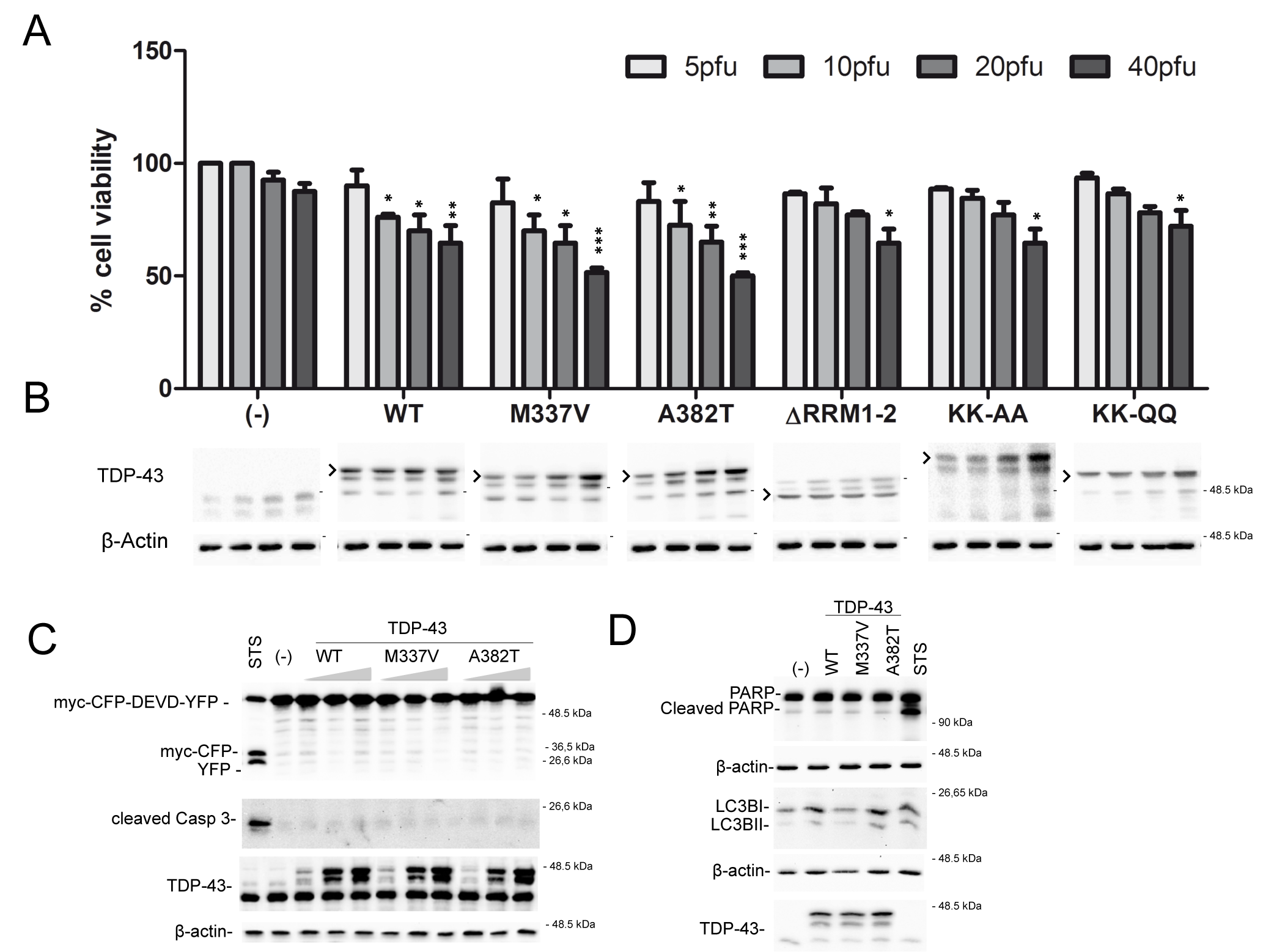

Supplement: Supplementary file 5 — Figure S4-rev [file 41419_2020_2580_MOESM5_ESM.tif]

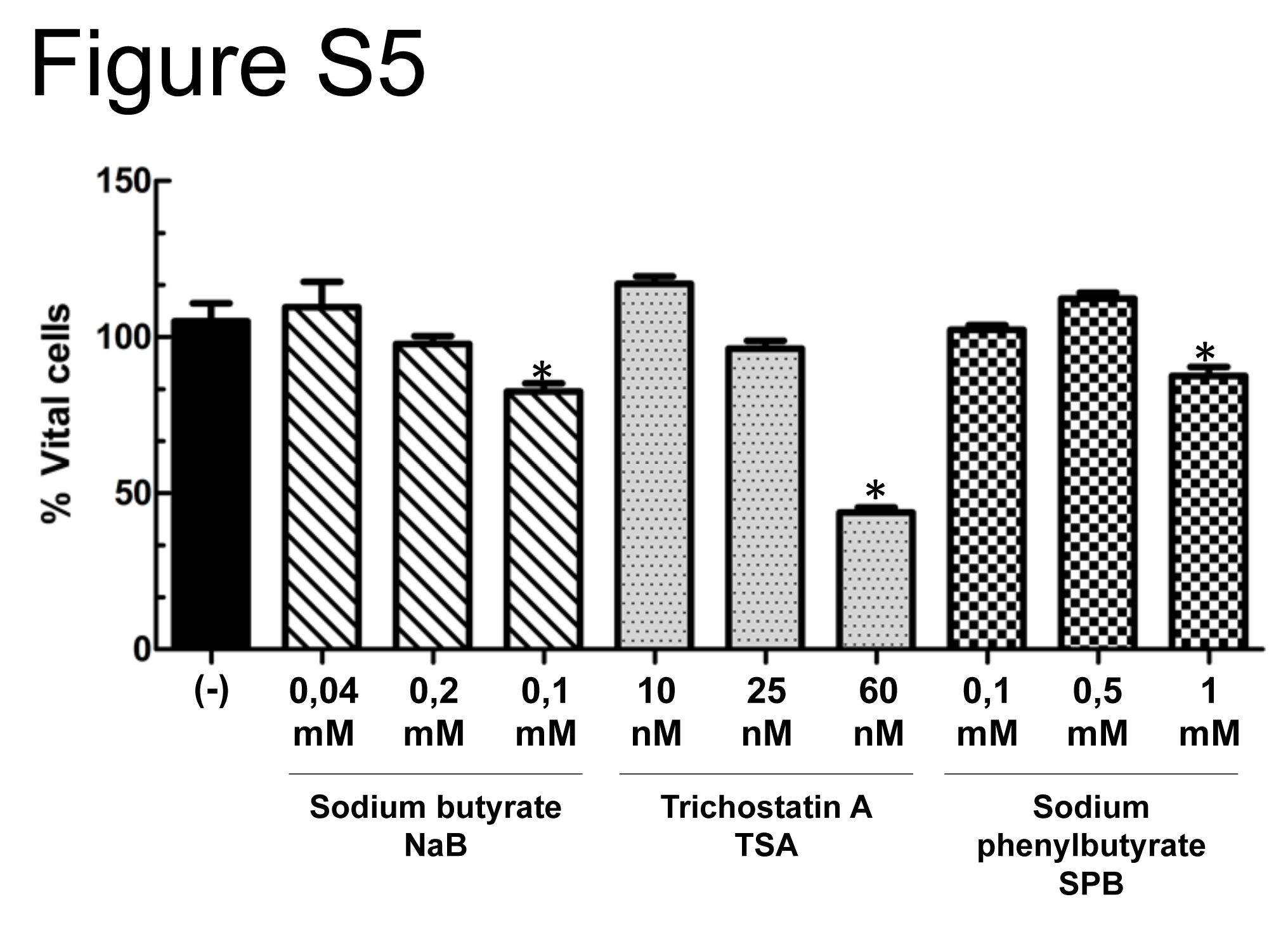

Supplement: Supplementary file 6 — Figure S5 [file 41419_2020_2580_MOESM6_ESM.tif]

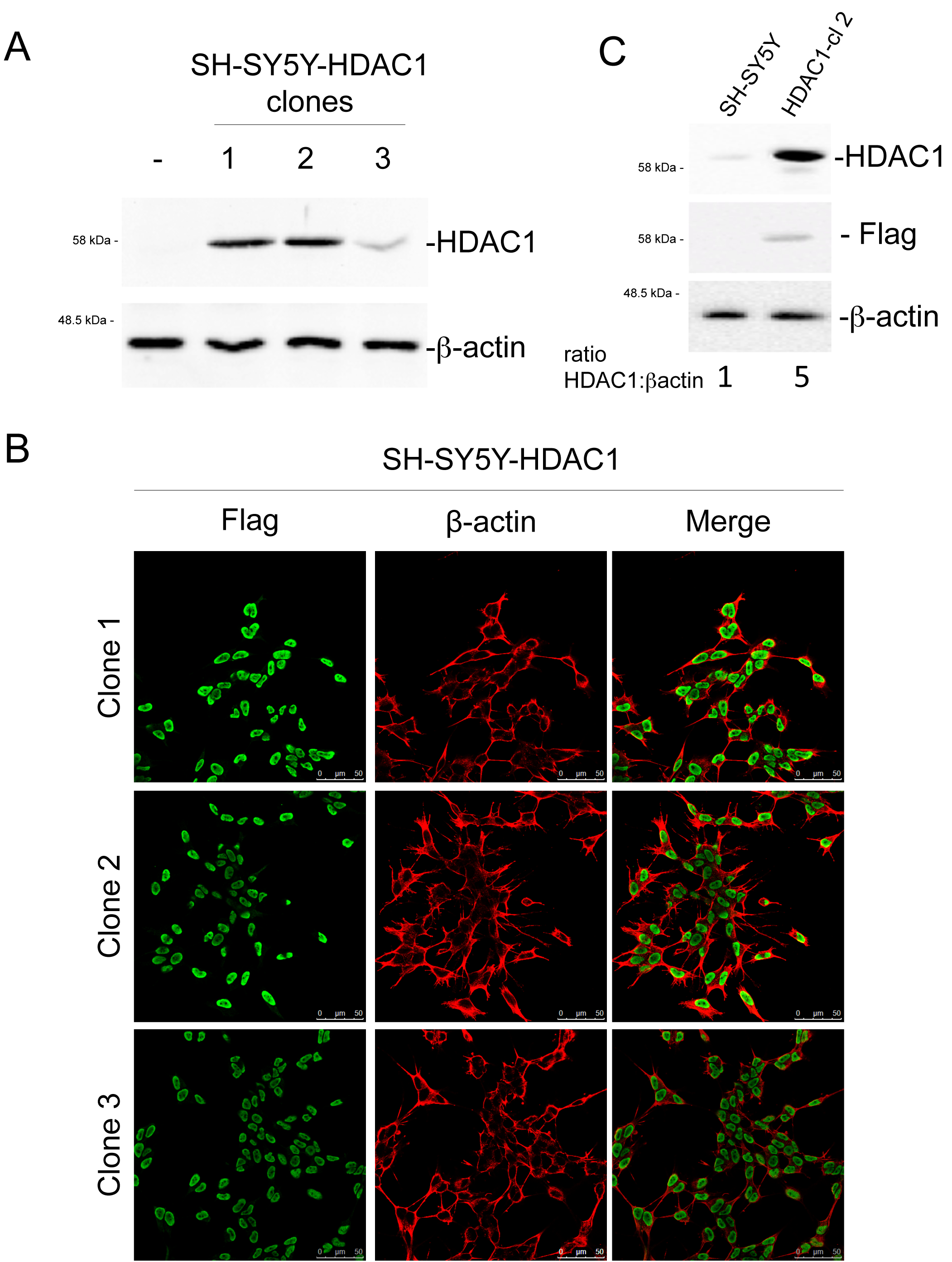

Supplement: Supplementary file 7 — Figure S6-rev [file 41419_2020_2580_MOESM7_ESM.tif]

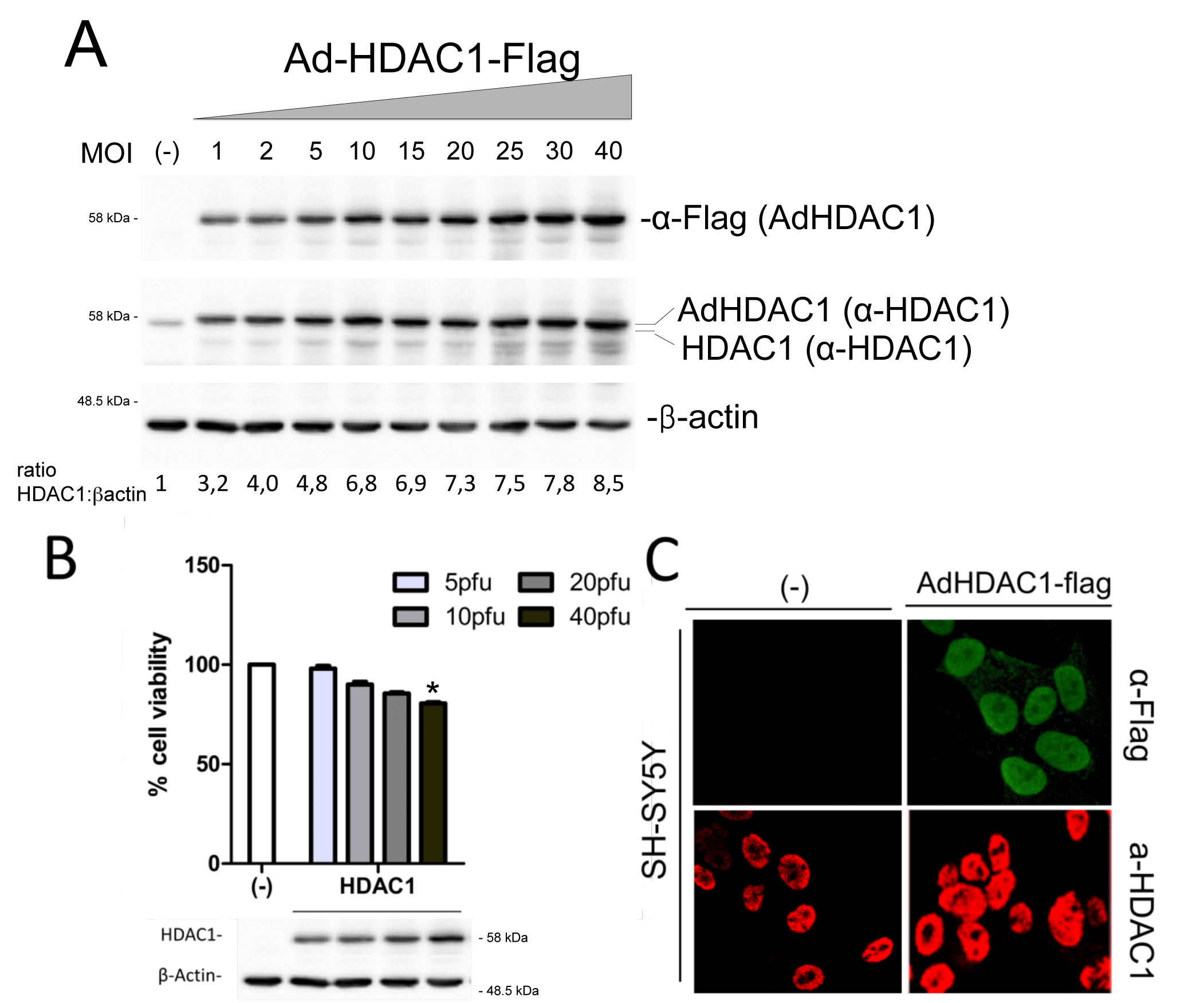

Supplement: Supplementary file 8 — Figure S7-rev [file 41419_2020_2580_MOESM8_ESM.tif]
